# Depth Discrimination for Low-Frequency Sources Using a Horizontal Line Array of Acoustic Vector Sensors Based on Mode Extraction

**DOI:** 10.3390/s18113692

**Published:** 2018-10-30

**Authors:** Guolong Liang, Yifeng Zhang, Guangpu Zhang, Jia Feng, Ce Zheng

**Affiliations:** 1 Acoustic Science and Technology Laboratory, Harbin Engineering University, Harbin 150001, China; liangguolong@hrbeu.edu.cn (G.L.); zhangyifeng1@hrbeu.edu.cn (Y.Z.); fengjia@hrbeu.edu.cn (J.F.); zhengce_uae@hrbeu.edu.cn (C.Z.); 2 Key Laboratory of Marine Information Acquisition and Security (Harbin Engineering University), Ministry of Industry and Information Technology, Harbin 150001, China; 3 College of Underwater Acoustic Engineering, Harbin Engineering University, Harbin 150001, China

**Keywords:** depth discrimination, horizontal line array, acoustic vector sensor, mode extraction

## Abstract

Depth discrimination is a key procedure in acoustic detection or target classification for low-frequency underwater sources. Conventional depth-discrimination methods use a vertical line array, which has disadvantage of poor mobility due to the size of the sensor array. In this paper, we propose a depth-discrimination method for low-frequency sources using a horizontal line array (HLA) of acoustic vector sensors based on mode extraction. First, we establish linear equations related to the modal amplitudes based on modal beamforming in the vector mode space. Second, we solve the linear equations by introducing the total least square algorithm and estimate modal amplitudes. Third, we select the power percentage of the low-order modes as the decision metric and construct testing hypotheses based on the modal amplitude estimation. Compared with a scalar sensor, a vector sensor improves the depth discrimination, because the mode weights are more appropriate for doing so. The presented linear equations and the solution algorithm allow the method to maintain good performance even using a relatively short HLA. The constructed testing hypotheses are highly robust against mismatched environments. Note that the method is not appropriate for the winter typical sound speed waveguide, because the characteristics of the modes differ from those in downward-refracting sound speed waveguide. Robustness analysis and simulation results validate the effectiveness of the proposed method.

## 1. Introduction

In underwater acoustics, how to discriminate between shallow sources and deep ones continues to be an active research area and is known as the problem of depth discrimination or classification. Source depth discrimination has many applications, including submarines, marine biology, and unmanned underwater vehicles [1].

There are two approaches to solving the problem of source depth discrimination. The first approach is based on the depth estimation. The traditional way to estimate source depth is matched-field processing, which requires simultaneous searches for the source range and depth using the range-depth ambiguity function. However, because of (i) the dependence of depth estimation on range estimation and (ii) the well-known environmental mismatch problem, estimating source depth is generally not robust [2,3,4]. To address this issue, Yang [4] proposed matched-mode processing (MMP) to estimate source depth independently of source range. Note that some modes are less sensitive to the mismatch environment, and the source depth can be estimated by matching those modes with corresponding replica modes. Therefore, MMP solves the environmental mismatch problem to a certain extent. To do so more effectively, the data-based matched-mode source localization method was proposed [5]. The main advantage of that method is that it is potentially free of the environmental mismatch problem because the wavenumbers and depth functions are estimated from data. However, the frequency of the moving source must be known a priori, which is difficult with passive sonar system. Next, unlike the first approach based on depth estimation, the second approach considers depth discrimination as a binary hypothesis test; examples include the matched-subspace method [6,7], the scintillation index (SI) [8], and the trapped energy ratio [9] to improve the robustness of depth discrimination.

Most of the aforementioned research was based on a vertical line array (VLA). However, we are interested herein in a horizontal line array (HLA) of acoustic vector sensors. Compared with a VLA, an HLA is more convenient to be placed and is more adaptable to its environment. The ability to resolve modes is determined by the effective horizontal aperture, which is the available horizontal aperture projected along the source azimuth. However, the required HLA is quite unrealistic in most practical cases, which has led to search for a new way to extract modes. Because the aperture of an HLA is limited, vector sensors are considered in source depth discrimination. A vector sensor measures important vector components of the acoustic field, such as the particle velocity along three orthogonal axes (the *x*, *y,* and *z* axes), which cannot be measured by a single scalar pressure sensor [10,11]; the radial particle velocity used herein is a combination of the *x* and *y* particle velocities. The vector information measured by a vector sensor can lead to better source depth discrimination results. Because it is difficult to estimate source depth effectively with a relatively short HLA, we instead consider the depth discrimination problem as a binary hypothesis test.

Because it is difficult to model the sound propagation accurately, the method used should be able to discriminate source depth even if the HLA is relatively short. Therefore, we propose herein a method for source depth discrimination that uses an HLA of vector sensors and is based on mode extraction for sources with low-frequency line spectra. We begin by using a normal mode model to describe the acoustic vector field. Next, we propose a mode-extraction method based on a TLS algorithm. Finally, according to modal amplitudes estimation, we propose depth-discrimination method based on the power percentage (PP) of low order modes. The originality of the present paper is reflected in two aspects, namely (i) the discriminator based on vector information and (ii) the proposed mode-extraction method. Because the energy leakage of other modes is considered besides that of the mode selected for extraction, the proposed mode-extraction method can reduce the required size of array aperture. Therefore, the proposed method can discriminate source depth robustly with a relatively short HLA.

This paper is organized as follows. In Section 2, we summarize the related literature on depth discrimination, which is considered as a binary hypothesis test. In Section 3, we propose a method for extracting modal amplitudes that can be used to discriminate source depth. In Section 4, we propose a method for source depth discrimination based on the PP of low-order modes. In Section 5, we evaluate the robustness of the method in different cases. We present a contrast experiment in Section 6, and we conclude the paper in Section 7.

## 2. Related Work

Various methods have been proposed recently for source depth discrimination problem, which is considered as a binary hypothesis test, and we summarize that literature briefly in this section. The methods can be divided into three groups based on the types of sensor array, namely source depth discrimination using (i) a VLA, (ii) an HLA, and (iii) vector sensors.

In the first group, Premus et al. proposed a matched subspace method to depth discrimination [6,7]. They used the difference in energy projected in the mode subspace between the shallow source and the deep one. Results of source depth discrimination with a discrete VLA of limited aperture are demonstrated in the SwellEX-96 experiment [7]. The SI can reflect the fluctuation of modal functions, which is closely related to source depth. Premus et al. discriminated source depth based on the value of SI [8]. However, SI can be used to discriminate source depth only if those ranges are sufficiently close, since it is influenced by the source-receiver range besides source depth. Considering the ill-posed problem of mode filtering when the water column is not well-sampled by the VLA, Conan et al. [9] proposed a robust method for discriminating the source depth supported by previous work introduced in [6,7,8]. When the VLA spans only 50% of the water column, that method outperforms those proposed in [6,7], as is evaluated by the receiver operating characteristic curve. The methods mentioned above in this paragraph are good at depth discrimination, but they require a VLA, something that is difficult to use with a mobile platform. In addition, it is difficult to stop a VLA from tilting, something that degrades the depth discrimination.

Compared with a VLA, an HLA is more conveniently placed and more adaptable to its environment. However, the fact that an HLA cannot sample in the depth direction makes depth discrimination with an HLA a complex task. Therefore, depth-discrimination methods based on an HLA usually underperform those based on a VLA. Yang obtained the wavenumber spectrum from the synthetic aperture beamforming and the discriminated depth from the structure of the wavenumber spectrum [1]. However, that method can be used only for cooperating sources, because it requires (i) the frequency of the original signal to be known a priori and (ii) a constant source speed. Based on the structure of the wavenumber spectrum, Reeder proposed an efficient approach for clutter-depth discrimination [12], which is used mainly for active sonar. Simulations showed that this technique could discriminate between a clutter source in the water column and one on the seabed. Premus et al. achieved source discrimination by using information about the source range, but it is difficult to obtain such information accurately with passive sonar [13]. The data results of the method demonstrated that the test statistic in [13] could discriminate between (i) a source towed at a depth of 100 m and (ii) the naturally radiated signatures of two surface ships in the same environment. Li separated the modes by using the beamforming in mode space [14], whereupon matched-mode processing was used to estimate the source depth. In simulations, that method performed well when the HLA was relatively long and the signal-to-noise ratio (SNR) was relatively high, but the performance decreased dramatically with a shorter HLA because of the low precision of mode extraction; ultimately, that method failed to discriminate the source depth effectively. In this paper, we discuss the case of a passive source and a relatively short HLA, a case to which none of the above methods can be applied directly.

In recent years, attention has been paid to vector sensors, which can obtain vector and scalar information synchronously and can be used for depth discrimination because of their acoustic intensity structure and capacity to suppress the isotropic noise. To discriminate source depth, Du used the local angle of the interference striations directly from the LOFAR diagram with the help of two-dimensional discrete Fourier transform to discriminate source depth [15]. Based on [15], Yang used double vertical passive vector sensors to improve the discrimination performance [16], using the active components of cross-spectrum of pressure and radial particle velocity to discriminate source depth. However, although the methods of both Du and Yang are capable of depth discrimination, they are based on the Pekeris waveguide, the use of which is impractical in an actual environment, especially for typical summer shallow water waveguide.

## 3. The Mode Extraction Method

In this section, we focus on a method of mode extraction for an HLA of vector sensors. In Section 3.1, we describe the acoustic vector field using the normal mode model. In Section 3.2, we illustrate the modal beamforming in the vector space. Based on the modal beamforming, we establish a relationship between the modal amplitudes and the modal beamforming output in the form of linear equations. In Section 3.3, we introduce the TLS algorithm to solve those linear equations and thus obtain the modal amplitude estimation. The modal amplitude estimation will be used to decision metrics, which are the core for depth discrimination.

### 3.1. Acoustic Vector Field

According to normal mode model, the acoustic vector field can be expressed as a finite sum of propagative modes. In the shallow waveguide, the signals on vector sensor *n* can be expressed as [17]

(1) $$p(r_{n},z_{s})=X\frac{e^{j\frac{\pi }{4}}}{\rho \sqrt{8\pi }}\sum_{m=1}^{M}\phi_{m}(z_{s})\phi_{m}(z_{r})\frac{e^{-\alpha_{m}r_{n}+jk_{m}r_{n}}}{\sqrt{k_{m}r_{n}}}+n_{p}(r_{n},z_{r})$$

(2) $$v_{r}(r_{n},z_{s})=X\frac{e^{j\frac{\pi }{4}}}{\rho^{2}\omega \sqrt{8\pi }}\sum_{m=1}^{M}\phi_{m}(z_{s})\phi_{m}(z_{r})k_{m}\frac{e^{-\alpha_{m}r_{n}+jk_{m}r_{n}}}{\sqrt{k_{m}r_{n}}}+n_{r}(r_{n},z_{r})$$

where $p(r_{n},z_{s})$ is the sound pressure and $v_{r}(r_{n},z_{s})$ is the radial particle velocity. $n_{p}(r_{n},z_{r})$ is the noise in the pressure channel, and $n_{r}(r_{n},z_{r})$ is the noise in the radial-particle-velocity channel. We assume that the noise terms in Equations (1) and (2) are independent and identically distributed zero-mean complex circular Gaussian processes and are channel-independent. We also assume that the noise is spherically isotropic, something that previous papers generally assume [10,18]. $z_{s}$ is the source depth, $z_{r}$ is the depth of sensor array, $r_{n}$ is the range between the source and sensor *n*, and ρ is the water density. ω is the angular frequency of source, X is the source complex amplitude at ω, M is the number of the propagative modes, $\alpha_{m}$ is the attenuation coefficient, $k_{m}$ is the horizontal wavenumber, and $\phi_{m}$ is the modal function of mode *m*.

### 3.2. Modal Beamforming in Vector Space with HLA

**Figure 1:**
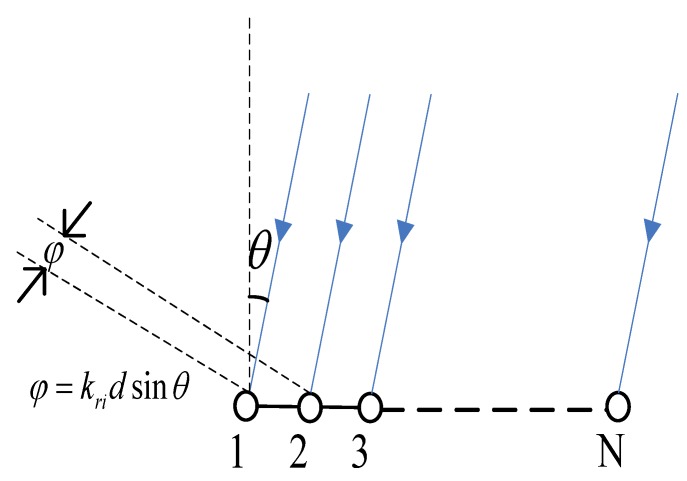
Phase difference normal mode *i*; θ is the azimuth of the source.

As shown in Figure 1, the HLA comprises of N sensors; the distance between two adjacent sensors is d, and the azimuth of the source is θ. Taking the sensor closest to the source (Sensor 1 in Figure 1) as the reference sensor (with distance $r_{1}=r$ to the source), the acoustic vector field is samples at ranges

(3) $$r_{n}=r+(n-1)d\sin\theta .$$

Moreover, with considering the far-field condition, the attention for geometric and viscous can be neglected along the HLA. Therefore, we can obtain the approximations for Equations (1) and (2): $r_{n}\approx r$ in the denominator and $e^{j\alpha_{m}r_{n}}\approx e^{j\alpha_{m}r}$ in the numerator. The sound pressure and radial particle velocity sampled by the HLA can be expressed as [11,14]

(4) $$p=s_{p}+n_{p}$$

(5) $$v_{r}=s_{r}+n_{r}$$

where $p={\left[p_{1}(r,z_{s})\cdots p_{N}(r,z_{s})\right]}^{T}$, $s_{p}={\left[s_{p1}(r,z_{s})\cdots s_{pN}(r,z_{s})\right]}^{T}$, $n_{p}={\left[n_{p1}(r,z_{r})\cdots n_{pN}(r,z_{r})\right]}^{T}$, $v_{r}={\left[v_{r1}(r,z_{s})\cdots v_{rN}(r,z_{s})\right]}^{T}$, $s_{r}={\left[s_{r1}(r,z_{s})\cdots s_{rN}(r,z_{s})\right]}^{T}$, $n_{r}={\left[n_{r1}(r,z_{r})\cdots n_{rN}(r,z_{r})\right]}^{T}$. $s_{pn}(r,z_{s})$, and $s_{rn}(r,z_{s})$ can be expressed as

(6) $$\begin{array}{ll} s_{pn}(r,z_{s}) & =X\frac{e^{j\frac{\pi }{4}}}{\rho \sqrt{8\pi }}\sum_{m=1}^{M}\phi_{m}(z_{s})\phi_{m}(z_{r})\frac{e^{jk_{m}[r+(n-1)d\sin\theta ]-\alpha_{m}r}}{\sqrt{k_{m}r}}\\ & =\sum_{m=1}^{M}d_{pm}(z_{s})\phi_{m}(z_{r})e^{jk_{m}(n-1)d\sin\theta } \end{array}$$

(7) $$\begin{array}{ll} s_{rn}(r,z_{s}) & =X\frac{e^{j\frac{\pi }{4}}}{\rho^{2}\omega \sqrt{8\pi }}\sum_{m=1}^{M}\phi_{m}(z_{s})\phi_{m}(z_{r})k_{m}\frac{e^{jk_{m}[r+(n-1)d\sin\theta ]-\alpha_{m}r}}{\sqrt{k_{m}r}}\\ & =\sum_{m=1}^{M}d_{vm}(z_{s})\phi_{m}(z_{r})e^{jk_{m}(n-1)d\sin\theta } \end{array}$$

where $d_{pm}$ and $d_{vm}$ are the modal amplitudes of pressure and radial particle velocity, respectively, and can be expressed as

(8) $$d_{pm}(r,z_{s})=X\frac{e^{j\frac{\pi }{4}}}{\rho \sqrt{8\pi }}\frac{e^{-\alpha_{m}r+jk_{m}r}}{\sqrt{k_{m}r}}\phi_{m}\left(z_{s}\right)\;$$

(9) $$d_{vm}(r,z_{s})=X\frac{e^{j\frac{\pi }{4}}}{\rho^{2}\omega \sqrt{8\pi }}k_{m}\frac{e^{-\alpha_{m}r+jk_{m}r}}{\sqrt{k_{m}r}}\phi_{m}\left(z_{s}\right).$$

Here, we review briefly the method proposed in [14], which extracts the modal amplitudes by modal beamforming. Figure 1 shows that the modal phase difference between different sensors varies with the wavenumber, where φ is modal phase difference *i*. Based on conventional beamforming, phase compensation is used to achieve in-phase stacking of modal amplitudes of specified order, while other components are offset more or less [14]. When the phase based on wavenumber *i* is compensated, only the order *i* term is in-phase stacking of modal amplitudes, while the others are offset more or less. Therefore, the modal amplitudes can be estimated by

(10) $$d_{pi}=\frac{1}{N\phi_{i}(z_{r})}\sum_{n=1}^{N}p_{n}(r,z_{s})e^{-jk_{i}(n-1)d\sin\theta }.$$

Note that the modal amplitudes can be estimated by modal beamforming with a relatively long HLA effectively [14]. How to estimate modal amplitudes with a relatively short HLA will be illustrated as follows. We deduce the relationship between modal amplitudes and beamforming output and extend the modal beamforming to the vector space. Beamforming output of mode *i* can be expressed as

(11) $$\begin{array}{c} b_{p}(i)=\frac{1}{N}\sum_{n=1}^{N}p_{n}(z_{s})e^{-jk_{i}(n-1)d\sin\theta }\\ =X\frac{e^{j\frac{\pi }{4}}}{\rho \sqrt{8\pi }}\sum_{m=1}^{M}\frac{\phi_{m}(z_{s})\phi_{m}(z_{r})e^{jk_{m}r}}{\sqrt{k_{m}r}}\frac{1}{N}\frac{\sin c[\frac{N}{2\pi }(k_{m}-k_{i})d\sin\theta ]}{\sin c[\frac{1}{2\pi }(k_{m}-k_{i})d\sin\theta ]}e^{j\frac{N-1}{2}(k_{m}-k_{i})d\sin\theta }+n_{\mathrm{bp}}(i)\\ =\sum_{m=1}^{M}d_{pm}(r,z_{s})f_{m}(k_{i})+n_{\mathrm{bp}}(i) \end{array}$$

where

(12) $$f_{m}(k_{i})=\frac{1}{N}\frac{\sin c[\frac{N}{2\pi }(k_{m}-k_{i})d\sin\theta ]}{\sin c[\frac{1}{2\pi }(k_{m}-k_{i})d\sin\theta ]}\phi_{m}(z_{r})e^{j\frac{N-1}{2}(k_{m}-k_{i})d\sin\theta },$$

and $n_{\mathrm{bp}}(i)$ is the noise of modal-pressure beamforming output *i*. When the HLA is relatively long, Equation (12) can be approximated as

(13) $$f_{m}(k_{i})\approx \left\{ \begin{array}{ll} \phi_{i}(z_{r}) & i=m\\ 0 & i\neq m \end{array}\right..$$

In this case, Equation (11) reduces to Equation (10), because except the selected mode (mode *i*), the other components can be ignored. When the HLA is relatively short, the approximation in Equation (13) no longer applies and therefore Equation (10) is a special issue of Equation (11). Similarly, we have

(14) $$b_{v_{r}}(i)=\frac{1}{N}\sum_{n=1}^{N}v_{\mathrm{rn}}(z_{r})e^{-jk_{i}(n-1)d\sin\theta }=\sum_{m=1}^{M}d_{vm}(r,z_{s})f_{m}(k_{i})+n_{\mathrm{bv}}(i)$$

where $n_{\mathrm{bv}}(i)$ is the noise in modal beamforming output *i* of the radial particle velocity. Here, we rewrite Equations (11) and (14) in matrix form as

(15) $$b_{p}=F\cdot d_{p}+n_{\mathrm{bp}}$$

(16) $$b_{v_{r}}=F\cdot d_{v_{r}}+n_{\mathrm{bv}}$$

where $b_{p}={\left[\begin{array}{ccc} b_{p}(1) & \ldots & b_{p}(M) \end{array}\right]}^{T}$, $b_{v_{r}}={\left[\begin{array}{ccc} b_{v_{r}}(1) & \ldots & b_{v_{r}}(M) \end{array}\right]}^{T}$, $d_{p}={\left[\begin{array}{ccc} d_{p1}(r,z_{s}) & \ldots & d_{pM}(r,z_{s}) \end{array}\right]}^{T}$, $d_{v_{r}}={\left[\begin{array}{ccc} d_{v1}(r,z_{s}) & \ldots & d_{vM}(r,z_{s}) \end{array}\right]}^{T}$, $n_{\mathrm{bp}}={\left[n_{\mathrm{bp}}(1)\;\cdots \;n_{\mathrm{bp}}(M)\right]}^{T}$, $n_{\mathrm{bv}}={\left[n_{\mathrm{bv}}(1)\;\cdots \;n_{\mathrm{bv}}(M)\right]}^{T}$, and $F=\left[\begin{array}{ccc} f_{1}\left(k_{1}\right) & \ldots & f_{M}\left(k_{1}\right)\\ \vdots & \ddots & \vdots \\ f_{1}\left(k_{M}\right) & \cdots & f_{M}\left(k_{M}\right) \end{array}\right]$.

From Equations (15) and (16), if matrix F is reversible, the modal amplitudes can be estimated directly as $d_{p}=F^{-1}\cdot b_{p}$ and $d_{v_{r}}=F^{-1}\cdot b_{v_{r}}$. However, a reversible F demands a very long HLA, and in practice the HLA cannot be as long as we require. Therefore, a novel TLS-based algorithm will be introduced to solve $d_{p}$ and $d_{v_{r}}$ for a singular or ill-conditioned F in Section 3.3.

### 3.3. Estimation of Modal Amplitudes Based on TLS

According to Equations (15) and (16), mode extraction can be viewed as the problem of solving the linear equations, where F is the coefficient matrix, $b_{p}$ and $b_{v_{r}}$ are constant vectors, and $d_{p}$ and $d_{v_{r}}$ are the unknown vectors. Usually, a least-square algorithm is used to solve the problem when the constant matrix contains an error Δb. Such an algorithm is effective when F is a robust matrix (with lower conditioning number). However, F may be ill-conditioned (with higher conditioning number) in the cases under consideration herein, and the error ΔF of the coefficient matrix may degrade the solution’s precision dramatically. Therefore, considering both Δb and ΔF, we introduce a TLS algorithm to solve the equations.

The basic principle of the present TLS algorithm is to solve the optimization problem as [2]

(17) $$\begin{array}{c} \underset{\Delta F,\Delta b_{p},d_{p}}{\min}{\left\|\left[\Delta F,\Delta b\right]\right\|}_{2}^{2}={\left\|\Delta F\right\|}_{2}^{2}+{\left\|\Delta b\right\|}_{2}^{2}\\ s.t.\;\left(F+\Delta F\right)\cdot d=b+\Delta b \end{array}$$

for which we use the approach given by [2] to calculate the estimation of d. The detailed steps are as follows.

First, we decompose the matrix B=[−b,F]. Using singular value decomposition B is decomposed as $B=U\Sigma V^{T}$.

Second, the number q of the main singular value is determined. Setting a threshold ε, the main singular value is selected when the normalized singular value is larger than ε, as ${\bar{\sigma }}_{i}=\frac{\sigma_{i}}{\sigma_{m}}\geq \varepsilon$, where $\sigma_{m}$ is the maximum main singular value.

Third, we calculate the estimation of d. Let $V_{1}$ comprise columns q+1 to M+1 of V as $V_{1}=\left[v_{q+1},v_{q+2},\cdots ,v_{M+1}\right]$, where $v_{q+1}$ is column *q* + 1 of V, ${\bar{v}}_{n}$ is row *n* of $V_{1}$, and let $V_{2}$ be the matrix, which is obtained by removing ${\bar{v}}_{1}$ from $V_{1}$. The estimation of d is then calculated by $\hat{d}=\frac{V_{2}{\bar{v}}_{1}^{H}}{{\bar{v}}_{1}{\bar{v}}_{1}^{H}}$, where $\hat{d}$ denotes the estimation of d.

## 4. Source Depth Discrimination

In this section, by analyzing the characteristics of modal depth function, we discriminate the source depth based on modal amplitude estimation. In Section 4.1, we briefly introduce the frameworks of depth-discrimination methods. In Section 4.2, we discuss the characteristics of the modal depth function in a typical summer waveguide. Based on the characteristics of modal depth function, we propose PP of low-order modes, which can be used to discrimination problem. In Section 4.3, we build the decision metrics and summarize the procedures for the source depth discrimination.

### 4.1. Frameworks of Depth-Discrimination Methods

**Figure 2:**
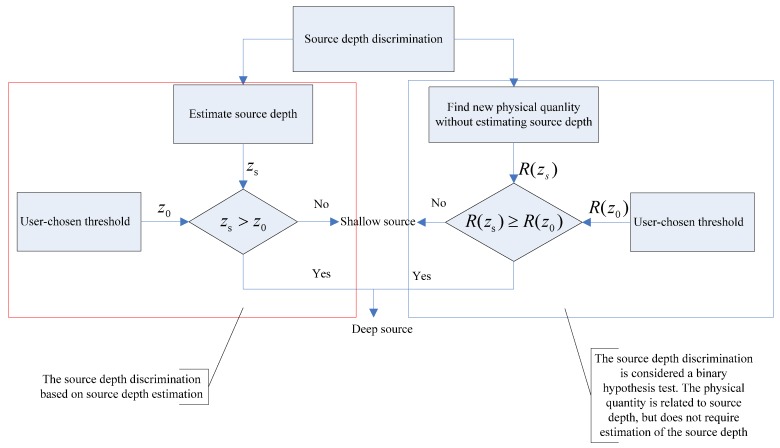
Procedural diagram for source depth discrimination. **Left** (red dashed box): depth discrimination based on depth estimation. **Right** (blue dashed box): depth discrimination based on binary testing hypotheses.

Here, we briefly introduce two frameworks of depth-discrimination approaches. In Figure 2, we divide depth-discrimination methods into two frameworks, namely one based on the source depth estimation (red dashed box on the left) and the other based on binary testing hypotheses (blue dashed box on the right).

For the framework based on the source depth estimation, the depth discrimination can be detailed as follows. Usually this framework estimates source depth first, then performs depth discrimination. After estimating the source depth $z_{s}$, a depth threshold $z_{0}$ (usually 5–10 m) is set to determine whether a source is shallow or deep. The source discrimination problem can be addressed as [9]

(18) $$\begin{array}{c} H_{0}:z_{s}\leq z_{0}\\ H_{1}:z_{s}>z_{0}. \end{array}$$

When $H_{0}$ is true, the source depth is smaller than $z_{0}$ and the source is distinguished as a shallow one. By contrast, $H_{1}$ corresponds to a deep one. Equation (18) is the physical basis for discriminating source depth based on estimated source depth.

For the other based on binary testing hypotheses, the depth discrimination methods are robust than the one based on depth estimation. The basic idea of this framework is to find another physical quantity related to $z_{s}$. In this way, source depth can be discriminated without estimating source depth $z_{s}$. We assume that the physical quantity can be expressed as $R(z_{s})$. Therefore, the source-discrimination model is written as [9]

(19) $$\begin{array}{c} H_{0}:R(z_{s})\leq \eta \\ H_{1}:R(z_{s})>\eta \end{array}$$

where η is the decision threshold. Herein, we propose the depth-discrimination method based on the second framework. In Equation (19), we see that the core of the method for discriminating source depth is selecting the decision metrics. In the next sub-section, we choose appropriate decision metrics by analyzing the characteristics of modal depth functions and propose PP of low-order modes to discriminate source depth.

### 4.2. Power Percentage of Low-Order Modes

**Figure 3:**
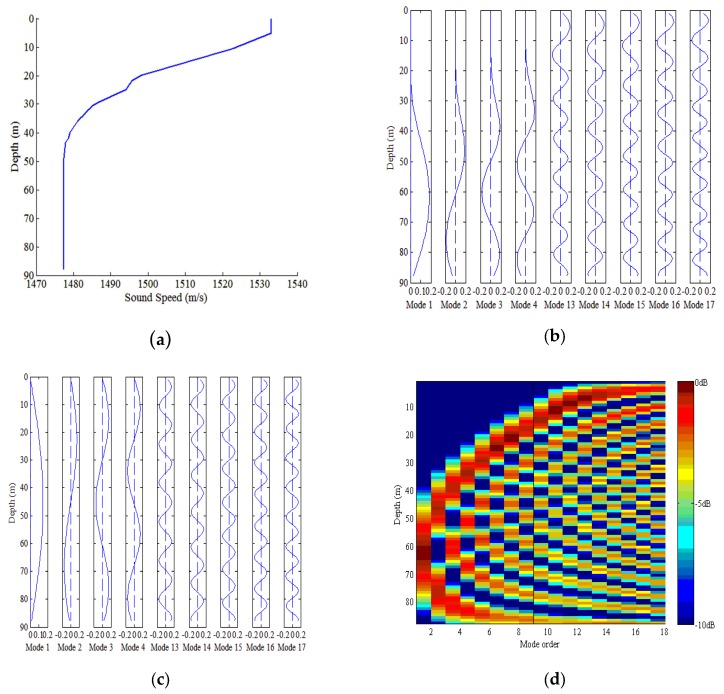
Characteristics of shallow water waveguide. (**a**) Sound speed profile (SSP) of typical summer shallow water waveguide. (**b**) Depth dependence of Mode Functions 1–4 and 13–17 for typical summer shallow water waveguide. (**c**) Depth dependence of Mode Functions 1–4 and 13–17 for typical winter shallow waveguide. (**d**) Normalized mode power for typical summer shallow water waveguide.

We consider an example of a typical shallow water waveguide with a downward-refracting sound speed to analyze the characteristics of modal depth functions. The water depth is 88 m. Figure 3a shows the sound speed profile (SSP) of a typical shallow water waveguide, and the corresponding modal depth functions for Modes 1–4 and 13–17 are shown in Figure 3b. The low-order modal depth functions such as Modes 1–4 present nearly sinusoidal functions below the transition point and decay evanescently in amplitude toward the surface above the transition point [6,7]. The transition point from sinusoidal to evanescent decay becomes shallower with the mode index [6,7]. For the high-order modes such as Modes 13–17 shown in Figure 3b, the transition point is at the surface, and only functions that are sinusoidal in amplitude are present [6,7]. This phenomenon of the trapping of low order modes is fundamental to the proposed method.

Note that the phenomenon described above pertains to the typical summer waveguide but not to a typical isovelocity winter waveguide. In the case of the Pekeris model, the mode function can be expressed as [17]

(20) $$\phi_{m}(z)=\sin(\gamma_{m}z)$$

where the vertical wavenumber $\gamma_{m}$ can be obtained from the dispersion equation, which is given by $\frac{\omega^{2}}{c^{2}(z)}=k_{m}^{2}+\gamma_{m}^{2}$; the studiers have been discuss how $k_{m}$ is obtained [1]. From Equation (20), we see that the mode function is the sinusoidal in the water waveguide regardless of whether the mode is of low order or high order. Therefore, the phenomenon described for a typical summer waveguide does not pertain to a typical isovelocity winter waveguide, as shown in Figure 3c. The proposed method is mainly suitable for a typical summer waveguide, rather than typical winter waveguide because we use the characteristics of the mode functions.

**Figure 4:**
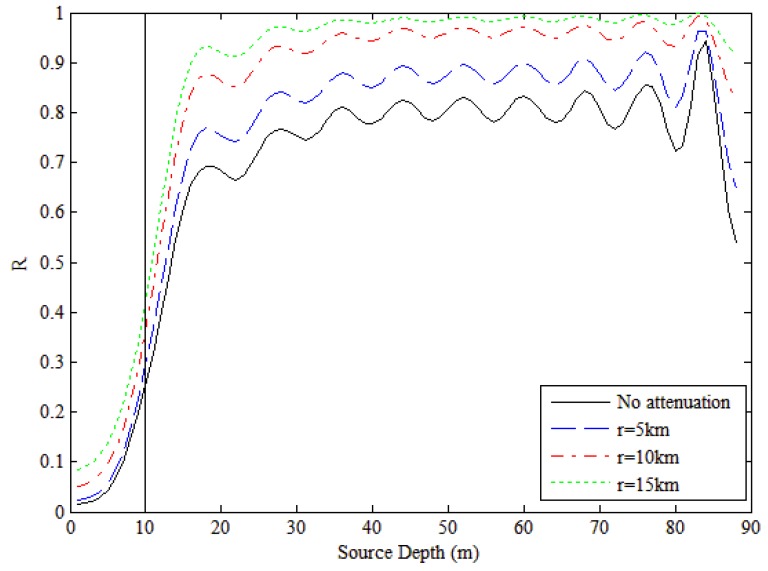
Evolution of power percentage (PP) of low order modes with source depth in shallow water waveguide for different source ranges. The vertical line indicates the discrimination depth *z*_0_ chosen in Section 5.

Figure 3d shows the normalized mode power for a typical summer shallow water waveguide, which indicates the value of ${\left|\phi_{m}(z)\right|}^{2}$. The normalized mode power is defined as $10\log_{10}\frac{{\left|\phi_{m}(z)\right|}^{2}}{P_{\max}}$, where $P_{\max}$ is the maximum value of ${\left|\phi_{m}(z)\right|}^{2}$ with all possible values of m and z. For shallow sources, the normalized power of the low-order modes is relatively low and that of high- order modes is relatively high. For the deep sources, the normalized power of the low-order modes and high-order modes has an approximate magnitude. That is to say, for shallow sources, the low-order modes contribute only a small percentage of the power among all the modes, whereas for deep sources, the low-order modes contribute a moderate percentage of power of all modes. Therefore, we consider discriminating the source by using the PP of the low-order modes. Given that the modal function cannot be obtained without knowing the depth, we use the correlation between the modal amplitudes of pressure and radial particle velocity to calculate the mode power, and the PP of low-order modes is given by

(21) $$R(z_{s})=\frac{\sum_{m=1}^{m_{0}}d_{pm}(r,z_{s})\cdot d_{vm}^{*}(r,z_{s})}{\sum_{m=1}^{M}d_{pm}(r,z_{s})\cdot d_{vm}^{*}(r,z_{s})}=\frac{\sum_{m=1}^{m_{0}}{\left|e^{-\alpha_{m}r}\phi_{m}\left(z_{s}\right)\right|}^{2}}{\sum_{m=1}^{M}{\left|e^{-\alpha_{m}r}\phi_{m}\left(z_{s}\right)\right|}^{2}}$$

where $R(z_{s})$ is defined as the PP of low-order modes. From Equation (21), we see $R(z_{s})$ also depends on the source range r. The curves in Figure 4 describe the characteristics of the PP. The black line represents the PP of the low-order modes without attenuation, which is independent of the source range, and the other three lines represent the PPs of low-order modes with different source ranges. For each line, the power percent increases dramatically with source depth below 20 m and then fluctuates slightly above 20 m. The depth threshold $z_{0}$ is usually selected from the range 5–15 m, and there is a one-to-one correspondence between PP and source depth in that range, allowing the corresponding PP to be determined directly. Herein, we take $z_{0}$ = 10 m (as shown in Figure 4). Because the source range influences the PP slightly, we can determine the PP threshold with assuming source range.

In the proposed method, the radial particle velocity is used to cancel the modes weighting by the horizontal wavenumber. If we use the pressure information only, then only the modal amplitude of pressure can be obtained. The decision metric in that case is given by

(22) $$R_{p}\left(z_{s}\right)=\frac{\sum_{m=1}^{m_{0}}d_{pm}(r,z_{s})\cdot d_{pm}^{*}(r,z_{s})}{\sum_{m=1}^{M}d_{pm}(r,z_{s})\cdot d_{pm}^{*}(r,z_{s})}=\frac{\sum_{m=1}^{m_{0}}\frac{{\left|e^{-\alpha_{m}r}\phi_{m}\left(z_{s}\right)\right|}^{2}}{k_{m}}}{\sum_{m=1}^{M}\frac{{\left|e^{-\alpha_{m}r}\phi_{m}\left(z_{s}\right)\right|}^{2}}{k_{m}}}.$$

Note that the atternuation term increases with mode order, whereas $k_{m}$ decreases. This means that the low-order modes have small weights, which is not the case in practice. In addition, because the noise of the radial particle velocity is independent of that of pressure, the proposed discriminator is better in suppressing noise than the discriminator in Equation (22). Therefore, using information about the radial particle velocity in the discriminator improves the ability to discriminate source depth. Herein, we focus on the uniform HLA of vector sensors; if the array is more complex, there might be a better way to take advantage of the particle velocity, an issue that we will be pursued in future work.

### 4.3. Source Discrimation Based on Mode Extraction

The previous analysis leads us to estimate the PP of low-order modes as a decision metric for source depth discrimination. Using the estimation of modal amplitudes ${\hat{d}}_{p}={\left[{\hat{d}}_{p1},\cdots ,{\hat{d}}_{pM}\right]}^{T}$ and ${\hat{d}}_{v}={\left[{\hat{d}}_{v1},\cdots ,{\hat{d}}_{vM}\right]}^{T}$ by the mode-extraction method,

(23) $$\hat{R}(z_{s})=\frac{\sum_{m=1}^{m_{0}}{\hat{d}}_{pm}(r,z_{s})\cdot {\hat{d}}_{vm}^{*}(r,z_{s})}{\sum_{m=1}^{M}{\hat{d}}_{pm}(r,z_{s})\cdot {\hat{d}}_{vm}^{*}(r,z_{s})}$$

where $m_{0}$ is the number of low-order modes. The low-order modes are defined as those with phase speeds lower than the maximum sound speed in the water column [9], and $m_{0}$ is determined by

(24) $$c_{p}(m_{0})<c_{\max},\;c_{p}(m_{0}+1)>c_{\max}$$

where $c_{p}(m)$ is the phase speed of the mode of order *m*, and $c_{\max}$ is the maximum sound speed. In this case, Modes 1–9 are the low-order modes and the rest are the high-order modes. The discrimination is performed by comparing the decision metrics to a decision threshold, and the decision threshold is set to $R(z_{0})$.

(25) $$H_{0}:\hat{R}(z_{s})\leq R(z_{0})\;H_{1}:\hat{R}(z_{s})>R(z_{0})$$

where $z_{0}$ is set 10 m. $H_{0}$ denotes the shallow source, and $H_{1}$ denotes the deep one.

**Figure 5:**
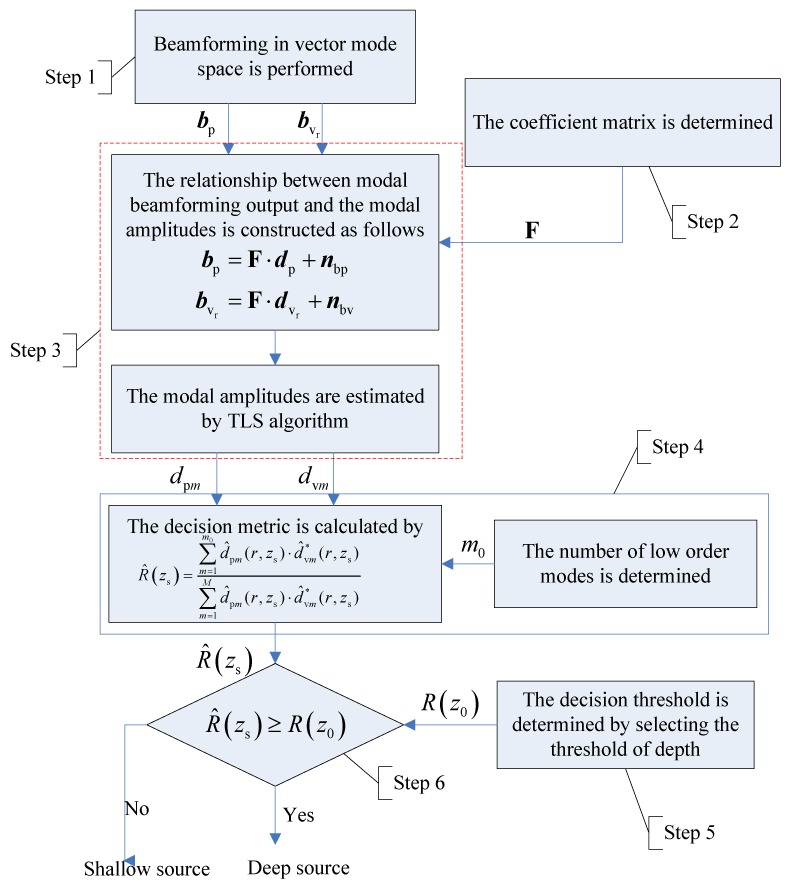
Diagram of source depth discrimination based on mode extraction.

To facilitate understanding of the proposed method, we summarize it diagrammatically in Figure 5 and with the following description. The proposed source-discrimination method based on mode extraction comprises the following steps.

Step 1. We implement beamforming in the vector mode space and obtain the output of sound pressure $b_{p}$ and radial particle velocity $b_{v_{r}}$ in Equations (11) and (14).

Step 2. The coefficient matrix F is constructed.

Step 3. We estimate the modal amplitudes ${\hat{d}}_{p}$ and ${\hat{d}}_{v_{r}}$ of sound pressure and vibration velocity in Equations (15) and (16) utilizing the TLS algorithm.

Step 4. We determine the threshold $m_{0}$ of low-order modes based on Equation (24) and calculate the decision metric according to Equation (21).

Step 5. We determine the depth threshold $z_{0}$ and calculate the decision threshold $\eta =R\left(z_{0}\right)$ with assuming source-receiver range.

Step 6. We perform source depth discrimination according to Equation (25). When $\hat{R}\left(z_{s}\right)\leq \eta$, the source is a surface source; otherwise, it is a deep one.

## 5. Robustness Analysis

In this section, we evaluate the robustness of the method. The simulation environment is established as follows. The SSP is shown in Figure 3a. We consider the bottom with a sound speed of 1650 m/s, a density of 1.76 g/cm^3^, and an attenuation of 0.8 dB/λ, where λ is the wavelength. The depth of the waveguide is 88 m, the HLA contains 100 sensors, and the distance between two adjacent sensors satisfies d=λ/2. The source is 5.01 km from the HLA with an azimuth of the source $\theta =60^{{}^{\circ}}$. The depth of the HLA is 50 m, whereas that of the source is selected randomly in each simulation with the variation range 0–88 m. The source’s frequency is 350 Hz and the SNR is 20 dB. From Equation (4), the SNR is defined as [9]

(26) $$\mathrm{SNR}=10\log_{10}\frac{{\left\|s_{p}\right\|}^{2}}{E\left\{{\left\|n_{p}\right\|}^{2}\right\}}.$$

The noise field can be expressed as a superposition of propagating plane waves from all possible directions [11]. To combine the pressure and radial particle velocity, both should be in the same units [10]. The magnitudes of pressure and particle velocity are related by p/ρc=v under the plane-wave approximation, where c is the sound speed [10]. Therefore, scaling the radial particle velocity by ρc allows us to define it in pressure units, which is the so-called pressure-equivalent radial particle velocity. Herein, we consider only the pressure and radial particle velocity. The power of the noise in channels of pressure and pressure-equivalent radial particle velocity should be identical [11]. The probability of the correct discrimination (PCD) is set to be the criteria. The PCD is defined as $\mathrm{PCD}=\frac{K_{c}}{K}\times 100\%$, where $K_{c}$ is the number of correct discriminations and K is the total number of experiment. We used 1000 Monte Carlo simulations (for each case) to evaluate the robustness of the method [19].

### 5.1. Influence of Number of the Sensor and the SNR on Performance

**Figure 6:**
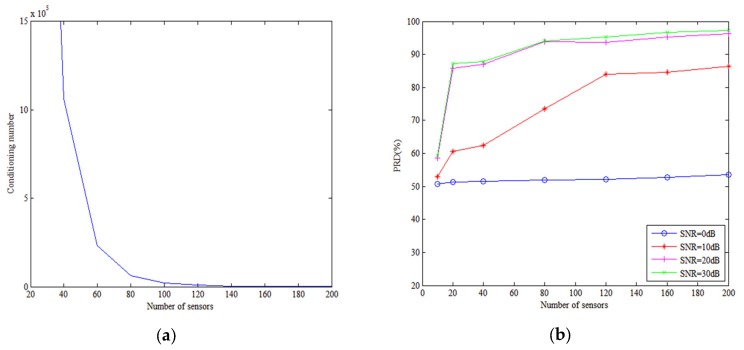
Conditioning number and performance evolution with the number of sensors for different SNRs. The tested number of sensors ranges from 10 to 200, and SNR is 0, 10, 20, and 30 dB. (**a**) Conditioning number of matrix F versus number of sensors. (**b**) PCD versus number of sensors.

**Figure 7:**
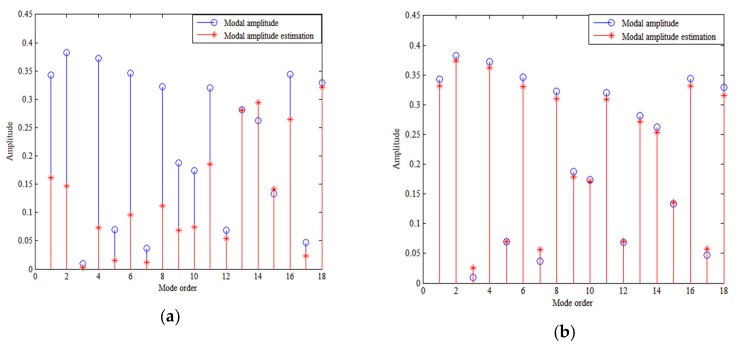
Modal amplitudes estimated by TLS algorithm with (**a**) 50 sensors and (**b**) 150 sensors. Depth of source and HLA are 50 m. SNR is 20 dB.

The number of sensors and the SNR are the two main influences on the PCD of the proposed method. Theoretically having a large number of sensors decreases the singularity of matrix F and increases the estimation precision of the modal amplitude, while a high SNR ensures that the received signal is of high quality and improves the PCD. Figure 6a shows the conditioning number of matrix F varies with the number of sensors. Figure 6b shows how the PCD varies with the number of sensors for different SNRs. Figure 7 shows the modal amplitude estimation results. When the SNR exceeds 10 dB (includes 10 dB), the method can discriminate the source depth with sufficient number of sensors. When the number of sensors is smaller than 100, the high conditioning number brings high error of modal amplitudes as shown in Figure 7a. The conditioning number decreases with the number of sensors as shown in Figure 6a. Then, the modal amplitude estimation precision increases with the number of the sensors. When the number of sensors is larger than 100, the modal amplitude estimation error is relatively small as shown in Figure 7b. It can be seen that when the number of sensors is 150, the modal amplitudes estimated are almost the same as the real ones. Increasing the number of sensors has little benefit to estimate modal amplitudes. Therefore, with fewer than 100 sensors, the PCD rises dramatically as the number of sensors is increased, stabilizing when there are more than 100 sensors.

When the SNR is 0 dB, the method fails to discriminate source depth. The noise level is relatively high compared with the power of low-order modes. In fact, the TLS-based method solves the equations by minimizing the norm of matrix error and noise. The excessive noise level makes the optimization problem no longer valid. The modal amplitude estimation method fails to work. Therefore, the PCD is around 50% in the case of the SNR is 0 dB.

### 5.2. Influence of HLA Depth on Performance

According to Equation (12), $f_{m}$ is related to the modal function $\phi_{m}\left(z_{r}\right)$. Therefore, the depth of the HLA can affect the singularity of matrix F and influence the PCD. For a relatively long HLA, Equation (12) can be approximated by Equation (13), allowing matrix F to be expressed as

(27) $$F=diag\{\phi_{1}(z_{r}),\phi_{2}(z_{r})\cdots \phi_{M}(z_{r})\}.$$

From Equation (15), the error of the pressure modal amplitude is ${\hat{d}}_{p}-d_{p}=F^{-1}b_{p}-d_{p}$ and the estimator bias $B(d_{p})=E\{{\hat{d}}_{p}-d_{p}\}=0$. The covariance matrix of ${\hat{d}}_{p}$ is

(28) $$K=E\{{\hat{d}}_{p}{\hat{d}}_{p}^{H}\}-E\{{\hat{d}}_{p}\}E{\left\{{\hat{d}}_{p}\right\}}^{H}=F^{-1}K_{\mathrm{nn}}{\left(F^{-1}\right)}^{H}$$

where $K_{\mathrm{nn}}$ is the spatial covariance of the noise vector and $K_{\mathrm{nn}}=\sigma_{n}^{2}I$, $\sigma_{n}^{2}$ is the power of the noise [20]. According to Equations (27) and (28), the covariance matrix is $K=diag\{\frac{\sigma_{n}^{2}}{\phi_{1}^{2}(z_{r})},\frac{\sigma_{n}^{2}}{\phi_{2}^{2}(z_{r})}\cdots \frac{\sigma_{n}^{2}}{\phi_{M}^{2}(z_{r})}\}$. When the HLA is located near the water surface, the power of the low-order modes is quite small and thus gives a very large error covariance. This does much to explain why the performance of the algorithm is not good when the HLA is near the water surface.

**Figure 8:**
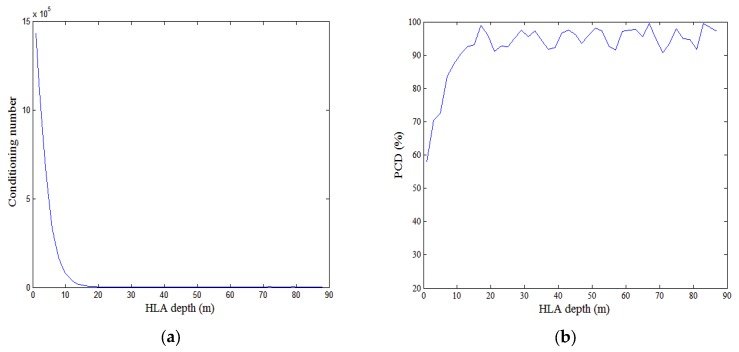
Conditioning number and PCD influenced by the depth of HLA. (**a**) Conditioning number of matrix F versus HLA depth. (**b**) PCD versus HLA depth.

**Figure 9:**
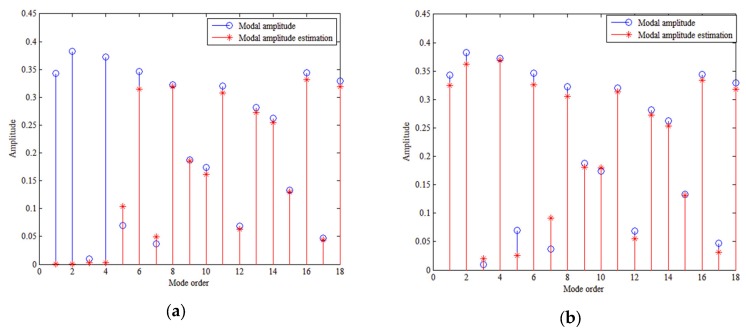
Modal amplitudes estimated by the TLS algorithm, when HLA depths are (**a**) 5 m and (**b**) 50 m. SNR is 20 dB.

For a short HLA, we use the conditioning number of F to analyze the performance. Figure 8a shows how the conditioning number of F varies with HLA depth. The PCD is plotted against depth in Figure 8b. The conditioning number has an effect on the modal amplitude estimation and PCD. When the HLA is located near the surface, the conditioning number is high and the estimation of low-order modal amplitudes is almost zero, as shown in Figure 9a. This means that the estimated value of PP is smaller than the real one. Therefore, a large number of deep sources are discriminated as shallow ones. In that case, the PCD is low. Figure 8a shows the conditioning number decreases dramatically with HLA depth when the depth is less than 10 m and stabilized once the depth exceeds 10 m. The accuracy of the modal amplitude estimation increases with the decreasing conditioning number. When the HLA depth exceeds 10 m, the modal amplitude estimation has a higher accuracy as shown in Figure 9b and the estimated value of PP is close to the real one. Therefore, the corresponding PCD increases with the HLA depth when the depth is less than 10 m. Despite oscillating, the PCD retains relatively high if the HLA is deep enough. For the simulation environment discussed in this section, an HLA depth of the 10 m ensures a high PCD (~90%).

### 5.3. Influence of Source Azimuth and the Azimuth Estimation Error Performance

The source azimuth and HLA aperture determine the effective array aperture. When the source is located at the end-fire direction of HLA, the effective array aperture achieves its maximum value, thereby benefiting modal beamforming. When the source is located at the abeam direction of the HLA, the best source azimuth estimation is obtained.

**Figure 10:**
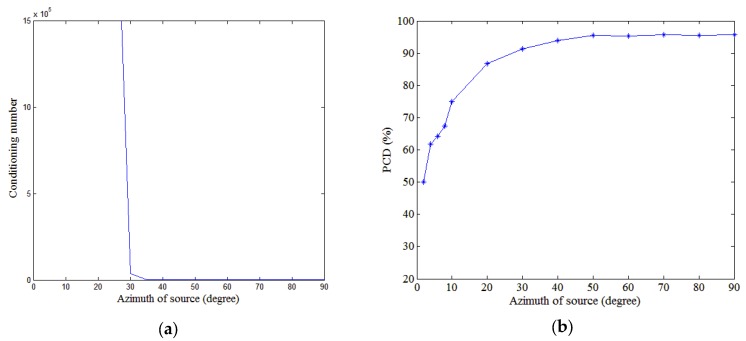
The conditioning number and the performance evolution with the source azimuth. (**a**) The conditioning number of matrix F versus the source azimuth. (**b**) PCD versus the source azimuth.

From Equation (12), the mode separation is related to $(k_{m}-k_{i})d\sin\theta$. When the source is located at the end-fire direction of HLA, $(k_{m}-k_{i})d\sin\theta$ achieves its maximum value without considering the error of the source azimuth estimation. In that case, the conditioning number of matrix F is low and the PCD is high. Otherwise, when the source is located at the abeam direction of HLA, $(k_{m}-k_{i})d\sin\theta$ is zero. Matrix F is completely noninvertible, and the method fails. Figure 10a shows the conditioning numbers of matrix F for sources with different azimuths, and Figure 10b shows the PCD for different azimuths. The conditioning number declines dramatically for azimuths below $30^{{}^{\circ}}$ and then stabilizes, so the PCD rises dramatically for azimuths below $30^{{}^{\circ}}$ and then stabilizes. In fact, we can explain the problem from the point of the effective array aperture. When the distance between two adjacent sensors is kept, the source azimuth and the number of sensors have a similar effect on the effective aperture. Therefore, the effect of the two on the performance of the proposed method is similar.

**Figure 11:**
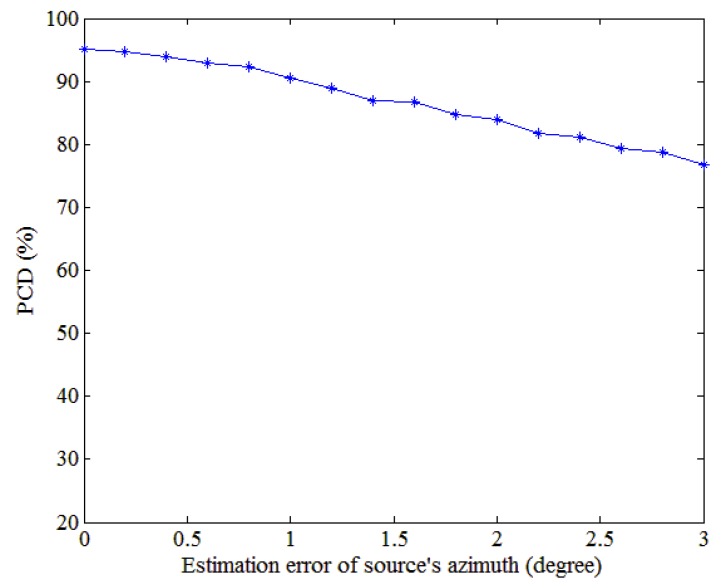
Performance evolution with the azimuth estimation error. The source azimuth estimation ranges from 0 to 3 degrees.

For a source azimuth of 60 degree, we consider source azimuth estimation errors of 0–3 degrees. Figure 11 shows how the PCD varies with the source azimuth estimation error in that range, from which the PCD clearly declines with the source azimuth estimation error. When the estimation error exceeds $1^{{}^{\circ}}$, the PCD is below 90%. Therefore, the method requires the azimuth to be estimated with high precision, which can be done by increasing the number of sensors.

### 5.4. Influence of the Frequency of Source Signal on Performance

**Figure 12:**
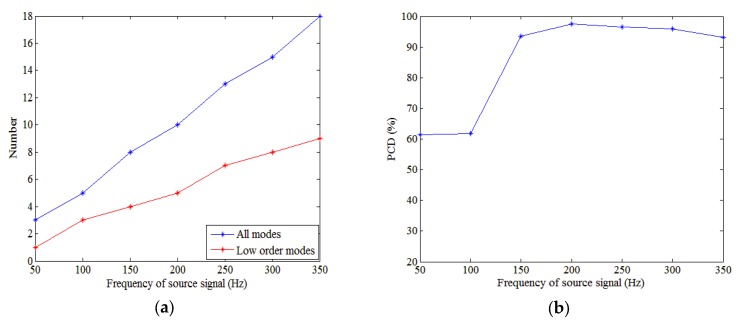
Number of modes and performance evolution with the source frequency. (**a**) Numbers of all propagative modes (blue) and low-order modes (red) versus frequency. (**b**) PCD versus the frequency of the source signal.

The frequency of the source signal influences the performance of the method for source depth discrimination. The number of low-order modes and all propagative modes are given in Figure 12a.

The effect of source frequency on PCD is illustrated in Figure 12b. This shows the PCD depends on the source frequency. As expected, the method is the most efficient at higher frequencies. Note that, at low frequency, the method has a relatively low PCD. Indeed, the small number of involved modes causes the method to lack effective information. For example, for a frequency of 50 Hz, the numbers of low-order modes and all propagative modes are one and three, respectively. The proposed method uses the PP of the modes. Because there is only one low-order mode, the PP is highly influenced by the performance of estimating this mode, thereby decreasing the robustness of the proposed method. Therefore, as shown in Figure 12b, the PCD is relatively low for frequencies below 100 Hz.

### 5.5. Performance in a Mismatched Environment

**Figure 13:**
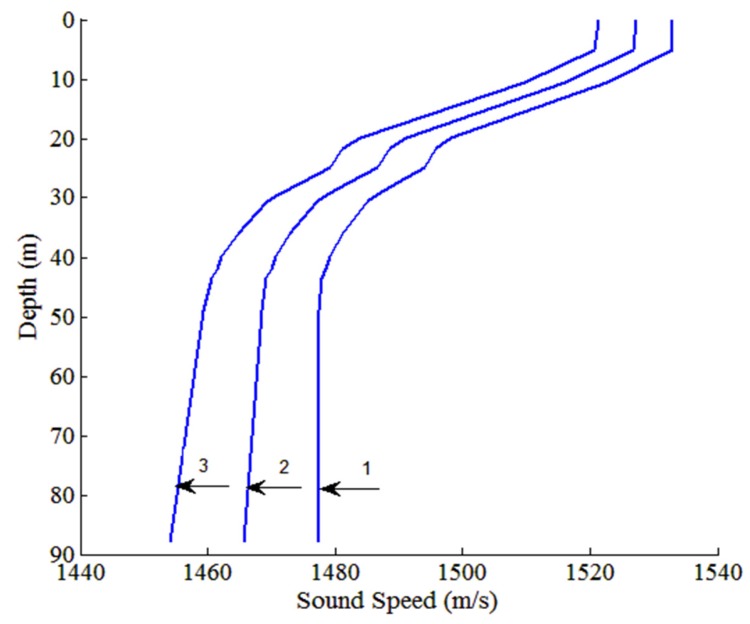
SSPs for different mismatched environments. Case 1 involves the actual SSP, Cases 2 and 3 involve mismatched SSPs. The error in the sound speed are 5.8–11.6 m/s in Case 2, and 11.6 m/s–23.2 m/s in Case 3.

In an actual situation, errors exist in parameters such as the SSP and sound speed, density, and attenuation of the bottom because of inaccurate measurements. Therefore, it is necessary to analyze the performance of the method in a mismatched environment. In this sub-section, the previous environmental model mentioned before is used to simulate data. Supposing a practical where this model is not available, the discriminator is built with a different environmental model (mismatch model). Therefore, the discriminator doesn’t match with data. We consider two environment types: with mismatched SSPs in the water column and with mismatched bottom parameters. In the first one, we focus on the cases of mismatched SSPs without any mismatch in bottom parameters. Figure 13 shows the SSPs of Cases 1–3, where Case 1 involves the real SSP (no mismatch) and Cases 2 and 3 are replaced by the real one adding SSP error [21]. The error of SSP is a linear function related to depth. In Case 2, the error in SSP are 5.8–11.6 m/s, which means the error in SSP at the surface is 5.8 m/s and at the bottom is 11.6 m/s. The error in SSP are 11.6–23.2 m/s in Case 3. Given that the three cases involve an offset in the SSP only, there is no major impact on modal structure. Adding a random perturbation to the SSP depth by depth, the additive perturbation is modeled as a zero-mean Gaussian process. The standard deviations of perturbation in Cases 4–6 are 3, 5, and 10 m/s, respectively. In the second one, the real SSP in the water column is kept, whereas the bottom parameters are replaced by the mismatched ones.

Table 1 lists the effect of environmental mismatch on the performance of discriminator, which include two environment types. From Table 1, we see that the performance deteriorates with the SSP error. The PCD decreases to 88.9% when the assumed SSP is in Case 2, which is still acceptable for depth discrimination. However, the PCD decreases further to 58.3% when the assumed SSP is in Case 3, causing the method to lose efficacy. The PCDs are 92.8% and 90.7% in Cases 4 and 5, respectively. Because of the large standard deviation of the perturbation, the modal structure changes with the increasing perturbation and the PCD decreases to 84.9%.

Besides the mismatched SSP, the method maintains a high PCD for mismatched sound speed, mismatched density, and mismatched attenuation of the bottom. The mismatch of bottom parameters has little effect on the number of modes and the modal structure. On the whole, the proposed method can tolerate an SSP error to some extent and is highly robust against mismatched environments.

## 6. Contrast Experiment

In this section, we compare the performance of the proposed method with those of two other existing depth-discrimination methods, namely the mode subspace projection (MSP) method [13] and the modal domain beamforming (MDB) method [14]. The simulation environments are the same as that in Section 5. The experiment is implemented with different sizes of HLA and in different mismatched environments, as described in Table 2. We consider fewer than 100 HLA sensors and a short HLA. Experiments 1 and 2 involve a short HLA. Experiments 3 and 5 involve mismatched environments. Experiment 4 involve a long HLA and no mismatched environments.

The experimental results are listed in Table 3. For a long HLA and a matched environment (Experiment 4), all three compared methods achieved high-precision depth discrimination, but the PCD of the proposed method was slightly lower than that of the MSP method, because the source range information was used in the MSP method. With a shorter HLA (corresponding to Experiments 1 and 2), the performance deteriorated but the proposed method maintained the lowest degree of deterioration among the methods. For the HLA with 50 sensors, the PCD of the MSP method decreased to 80%, the MDB method failed, and the PCD of the proposed method remained above 90%. Because the proposed mode extraction method is suitable for a short HLA. With mismatched environment (Experiments 3 and 5), the PCDs of the MSP and MDB methods decreased by around 11%, whereas that of the proposed method decreased by around only 6%, indicating that our method was one that was most robust against a mismatched environment. Overall, the proposed method surpassed the other two methods for a short HLA or a mismatched environment.

## 7. Conclusions

In this paper, we addressed the problem of depth discrimination for low-frequency sources using an HLA of vector sensors. First, we derived an expression for beamforming in the vector mode space and established linear equations related to the modal amplitudes. We then solved linear equations by introducing a TLS algorithm, thereby estimating the modal amplitudes in high precision. Finally, we discriminated the source depth based on the extracted modal amplitudes.

To evaluate the performance of the proposed method, we performed robustness analysis and contrast experiment. The analysis showed that the proposed method can achieve a high PCD with more than 100 sensors, an HLA depth in excess of 10 m, a source azimuth in excess of 30 degrees, a source azimuth estimation error less than 1 degree, and a frequency above 100 Hz. Moreover, it indicated that the proposed method is highly robust against a mismatched environment. In the contrast experiment, we compared (i) the proposed method, which computes the PP in the vector mode space and extracts the modes by means of the TLS algorithm, with two other methods, namely (ii) MSP, which uses projections and discriminate depth in sensor space, and (iii) MDB, which discriminates source depth based on MMP. The results of contrast experiment showed that the proposed method performs better for a short HLA and mismatched SSP compared with MSP and MDB. This is because (i) working in the mode space makes the metrics less sensitive to any mismatch and (ii) the mode extraction method used herein is more suitable to the case of a short HLA.

In the present work, the method was based on a range-independent environment. Therefore, the feasibility of the method should be assessed for a range-dependent environment, which is closer to the actual SSP environment. We also intend to investigate how to reduce the dependence on prior knowledge and how to use the vertical particle velocity effectively.

## Figures and Tables

**Table 1:** Probability of correct discrimination (PCD) in different mismatched environments.

SSP Error	Error in Sound Speed of Bottom (m/s)	Error in Density of Bottom (g/cm^3^)	Error in Attenuation of Bottom (dB/*λ*)	PCD
Case 1	0	0	0	95.6%
Case 2	0	0	0	88.9%
Case 3	0	0	0	58.3%
Case 4	0	0	0	92.8%
Case 5	0	0	0	90.7%
Case 6	0	0	0	84.9%
Case 1	0	0.1	0	94.5%
Case 1	0	−0.1	0	94.9%
Case 1	0	0	−0.16	95.4%
Case 1	0	0	0.24	95.3%
Case 1	10	0	0	94.5%
Case 1	−10	0	0	94.5%

**Table 2:** Experimental environments.

Experiment	Number of Sensors	SSP Error
1	50	Case 1
2	100	Case 1
3	100	Case 2
4	500	Case 1
5	500	Case 2

**Table 3:** Experimental results.

Experiment	PCD of MSP Method	PCD of MDB Method	PCD of Proposed Method
1	80.3%	52.2%	90.3%
2	90.7%	73.2%	95.3%
3	79.8%	65.5%	88.9%
4	98.8%	90.6%	98.1%
5	94.1%	85.0%	97.6%

## References

[1] Yang T.C. (2015). Source depth estimation based on synthetic aperture beamfoming for a moving source. J. Acoust. Soc. Am..

[2] Baggeroer A.B., Kuperman W.A., Mikhalevsky P.N. (1993). An overview of matched field methods in ocean acoustics. IEEE J. Ocean. Eng..

[3] Wang Q., Wang Y.M., Zhu G.L. (2017). Matched field processing based on least squares with a small aperture hydrophone array. Sensors.

[4] Yang T.C. (1987). A method of range and depth estimation by modal decomposition. J. Acoust. Soc. Am..

[5] Yang T.C. (2014). Data-based matched-mode source localization for a moving source. J. Acoust. Soc. Am..

[6] Premus V.E., Backman D. A matched subspace approach to depth discrimination in a shallow water waveguide. Proceedings of the Conference Record of the Forty-First Asilomar Conference on Signals, Systems and Computers.

[7] Premus V.E., Ward J., Richmond C.D. Mode filtering approaches to acoustic source depth discrimination. Proceedings of the Conference Record of the Thirty-Eighth Asilomar Conference on Signals, Systems and Computers.

[8] Premus V.E. (1999). Modal scintillation index: A physics-based statistic for acoustic source depth discrimination. J. Acoust. Soc. Am..

[9] Conan E., Bonnel J., Chonavel T., Nicolas B. (2016). Source depth discrimination with a vertical line array. J. Acoust. Soc. Am..

[10] Paulo F., Paulo S., Sérgio M.J. (2018). Acoustic pressure and particle velocity for spatial filtering of bottom arrivals. IEEE J. Ocean. Eng..

[11] Malcolm H., Arye N. (2001). Acoustic vector-sensor correlations in ambient noise. IEEE J. Ocean. Eng..

[12] Reeder D.B. (2014). Clutter depth discrimination using the wavenumber spectrum. J. Acoust. Soc. Am..

[13] Premus V.E., Helfrick M.N. (2013). Use of mode subspace projections for depth discrimination with a horizontal line array: Theory and experimental results. J. Acoust. Soc. Am..

[14] Li P., Zhang X.H., Fu L.F., Zeng X.X. (2017). A modal domain beamforming approach for depth estimation by a horizontal array. Acta Phys. Sin..

[15] Du J.Y., Zheng Y., Wang Z.Q., Cui H., Liu Z.W. Passive acoustic source depth discrimination with two hydrophones in shallow water. Proceedings of the OCEANS.

[16] Yang G., Yin J.W., Yu Y., Shi Z.H. (2016). Depth classification of underwater targets based on complex acoustic intensity of normal modes. J. Ocean Univ. China (Ocean. Coast. Sea Res.).

[17] Sullivan E.J. The Use of P-V sensors in passive localization. Proceedings of the OCEANS.

[18] Zhong X.H., Permkumar A.B. (2012). Particle filtering approaches for multiple acoustic source detection and 2-D direction of arrival estimation using a single acoustic vector sensor. IEEE Trans. Signal Proc..

[19] Sun S.B., Liang G.L. (2018). ISAR imaging of complex motion targets based on Radon transform cubic chirplet decomposition. Int. J. Remote Sens..

[20] Buck J.R., Preisig J.C., Wage K.E. (1998). A unified framework for mode filtering and the maximum a posteriori mode filter. J. Acoust. Soc. Am..

[21] Feuillade C., Balzo D.R.D., Rowe M.M. (1989). Environmental mismatch in shallow-water matched-field processing: Geoacoustic parameter variability. J. Acoust. Soc. Am..

